# Seamless Copy–Move Replication in Digital Images

**DOI:** 10.3390/jimaging8030069

**Published:** 2022-03-10

**Authors:** Tanzeela Qazi, Mushtaq Ali, Khizar Hayat, Baptiste Magnier

**Affiliations:** 1 Department of Information Technology, Hazara University Mansehra, Mansehra 21120, Pakistan; tn.kazi@gmail.com (T.Q.); mushtaq@hu.edu.pk (M.A.); 2 Department of Mathematical and Physical Sciences (DMPS), College of Arts and Sciences (CAS), University of Nizwa, Nizwa 616, Oman; 3 EuroMov Digital Health in Motion, Univ Montpllier, IMT Mines Ales, 30100 Ales, France; baptiste.magnier@mines-ales.fr

**Keywords:** discrete wavelet transform (DWT), copy–move replication, image manipulation, image tampering, image forgery, frequency domain, edge detection

## Abstract

The importance and relevance of digital-image forensics has attracted researchers to establish different techniques for creating and detecting forgeries. The core category in passive image forgery is copy–move image forgery that affects the originality of image by applying a different transformation. In this paper, a frequency-domain image-manipulation method is presented. The method exploits the localized nature of discrete wavelet transform (DWT) to attain the region of the host image to be manipulated. Both patch and host image are subjected to DWT at the same level *l* to obtain 3l+1 sub-bands, and each sub-band of the patch is pasted to the identified region in the corresponding sub-band of the host image. Resulting manipulated host sub-bands are then subjected to inverse DWT to obtain the final manipulated host image. The proposed method shows good resistance against detection by two frequency-domain forgery detection methods from the literature. The purpose of this research work is to create a forgery and highlight the need to produce forgery detection methods that are robust against malicious copy–move forgery.

## 1. Introduction

In image manipulation, composition, editing, tampering, forgery, or fakery, the ultimate victim is the integrity and authenticity of the image. The usage spectrum is broad, with aesthetics on one extreme and malicious intents (such as blackmailing and character assassination) on the other. Readily available software such as Adobe Photoshop, GIMP, or even XnView has further escalated the matter. No matter how noble intentions are, while introducing any innovation to manipulate images, the stakes of negativity are always high. The burden to deal with such negativity shifts is on the forensic analyst. Aptly described as an “arms race” in [1], this competition between manipulator and forensic analyst may never end.

As “a picture is worth a thousand words”, authenticity and trustworthiness of images are legally and socially very important. For security purposes, several approaches were developed, broadly categorized as active and passive methods. Research in passive methods is receiving increasing attention because of the limitations in the active counterparts, especially its reliance on watermarks: it should be embedded at the time of image acquisition that requires a specially equipped camera or devices and most of the watermarks degrade image quality while manipulating the image for the insertion of watermark or related processing [2,3].

Blind image forensics only deals with the image under investigation without expecting any additional side information. It thus plays a vital role in many areas, including medical imaging, news reporting, criminal or court room investigations, insurance claim investigations, sports, and intelligence services [1,2,3]. In passive approaches, the image is forged through some sophisticated software or image manipulation techniques, where the goal is imperceptibility and not leaving any observable traces indicative of fakery, such as motion blur, broken edges, or edge inconsistencies.

In this paper, we propose a passive copy–move image manipulation method that exploits the localized nature of the discrete wavelet transform (DWT). The idea is to pass the image to the frequency domain and subject each resultant individual sub-band to copy/move image manipulation. We thus transform both patch and host image into the DWT domain, and try to paste the sub-bands of the patch on corresponding sub-bands of the host. This is followed by the inverse DWT to obtain the manipulated image to achieve homogeneity between the patch and its new environment in the host; the inverse DWT operation should be vigorous enough to ensure the required scrambling in order to suppress any side effects. Indeed, the result should be the seamless manipulation of the host to obtain a forged image that bears no forensic evidence of tampering.

**Figure 1:**
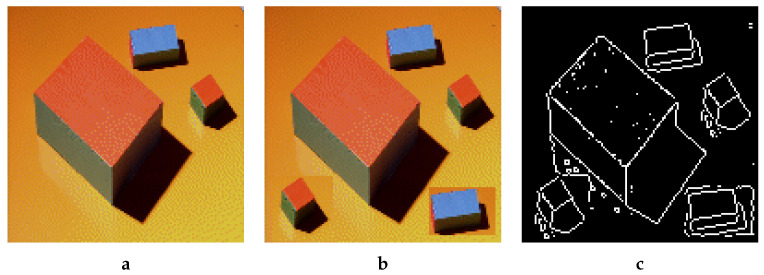
(**a**) Original, (**b**) Forged, (**c**) Sobel edge detection. Example of forged image in (**b**) with its tied Sobel edge detection. A simple copy of some parts in the original image may create rectangles in the resulting image.

Our use of DWT is based on the fact that, even with skilled and careful copy–move forgery, the borders of the copied patch in forged image may show edge inconsistencies. Hence, a simple edge detection algorithm should be enough to catch a forgery, no matter how much it was attempted to smoothen the transition from host image to the copied patch, as illustrated in Figure 1. Even postprocessing with operations such as patch rotation can be countered with good edge detection algorithms, as some are sensitive to even very low degrees of rotation (see details in [4]). To counter edge detection and other similar techniques, we aim to dilute the potential artifacts by pasting the wavelet transformed sub-bands of the patch in the corresponding sub-bands of the host and then applying inverse DWT to the latter, i.e., the tampered host sub-bands.

To ascertain the effectiveness of our methods, we chose two very recent frequency-domain forgery detection methods from the literature [5,6] as reference. Results suggest that these two methods were not that successful in detecting manipulation, a fact recognized by the creators of the two methods.

The rest of the paper is arranged as follows. Section 2 presents a concise survey of the related literature. This is followed by the presentation of the proposed method in Section 3. Simulation results are illustrated in Section 4 in detail. Section 5 concludes the paper.

## 2. Literature Review

In this section, image forgery and forgery detection methods are presented. In addition, we dedicate a separate section to frequency-based detection methods, with special reference to DWT. The purpose of this research work was to generate an image forgery that had no visual clues or tampering evidence, and to check the strength of the forged image created by our own proposed method with existing state-of-the-art methods.

### 2.1. Image Manipulation

Image manipulation or image forgery encompasses any technique that may be used to manipulate an image [1]. It may be carried out using either active or passive approaches [2,3]. Active approaches are mostly concerned with data hiding techniques, such as digital watermarks and signatures, where prior information is considered to be essential and integral to the process. Passive or blind approaches do not require any prior information about the original image [3,7,8], and the analyzer has just the final product at their disposal. A forensic expert prefers the term “image forgery” over “image manipulation” and would classify it as [9,10,11]:

1. Copy/move forgery or cloning where the patch comes from the host image; detection is relatively hard because source and destination image is the same, and color and noise are the same for the rest of the image.
2. Image splicing where the patch comes from a different image than the host does, and detection is comparatively easy because source and destination are from different images or set of images.
3. Image retouching, which encompasses a wide spectrum of techniques to enhance the visual appearance of the image at hand, and this is the least pernicious type of forgery, widely used by magazine photography editors.

In technical terms, the former two, i.e., copy/move and splicing, form the basis of object transferring. Many object transfer techniques exist [1], but the following are the most popular:

- Cut-out: patch boundaries should be well-defined, and the objective is for the contours to be seamless in a variety of ways, e.g., RepSnapping [12] and Intelligent Scissors [13]. Such manipulations are not concerned with the original environment of the patch and are thus easy to detect.
- Alpha matting: soft extraction of a foreground [14] that is similar to cut-out but uses alpha-transparency adjustments between original and destination images to dilute boundaries.
- Gradient domain techniques: have the goal of blending the gradient of the patch with that of the host. Among these, the most popular ones are perhaps those based on interpolations through Poisson equations for gradient match, which is a technique referred to as Poisson editing [15] or seamless cloning [16].
- Laplacian pyramids were also employed in many works during the blending process [17,18].

In DWT, Hayat et al. [19] presented two transform domain methods to seamlessly stitch satellite image tiles of heterogeneous resolutions. One is local, and each constituent DWT domain tile of the view is treated at sub-band level with horizontal, vertical, and radial smoothing on the basis of its locale in the tessellation. The second method assumes the view field to be of a sliding window containing parts of the tiles from the heterogeneous tessellation. The window is subjected to DWT domain mosaicking and smoothing. The last step in both the methods is overall inverse DWT.

Except for image manipulation by easily available software, the authors in [20] proposed a forgery method for an experiment on detection techniques that showed good resistance to forgery detection. In this method, the patch image mask is produced from the host image and pasted on the host image to obtain a forged image.

In [8], the process of image forgery is described as the selection of the region of interest (ROI), the transformation and composition of image fragments, and some necessary postprocessing on the final image. The process usually begins with extracting the fragments and fusing the transformed image fragments into another image using different techniques, such as matting/pasting for coherent looking composition. The method claims to visually produce no tampering evidence in the face of existing techniques in forgery detection. In [21] different technologies and tampering techniques were described for digital images that are difficult to detect. According to the authors, the problem of establishing the authenticity and reality of digital photography is now more complex and challenging.

### 2.2. Forgery Detection

The literature is replete with surveys on the forgery detection literature [2,3,22,23]. Copy–move forgery detection methods can be broadly classified into three categories:

- block-based;
- key point based; and
- hybrid techniques.

Block-based methods are highly desirable for locating forged regions via block-by-block matching. An example could be the technique outlined in [24], which divides a suspect image into overlapping circular blocks. This is followed by the extraction of geometric transform-invariant features by the application of polar complex exponential transform (PCET), which is then subjected to singular-value decomposition (SVD) for dimensionality reduction in parallel to computing a histogram-based similarity threshold that is employed for a block matching process. The method claims to be very successful against forgeries resisting rotation and scaling. Block-based methods demonstrate poor accuracy when some postprocessing is performed, e.g., noise addition, blurring and compression or contrast changing, or a combination. In addition, they are not robust to geometric transformations and involve high computational cost [23].

Key-point-based methods are well-suited for quick decisions about a suspicious image; they have low computational cost and remarkable performance with respect to memory requirements. An example is [25], which first approximates suspected parts through keypoint estimation based on iterative Delaunay triangle matching. Keypoint pairs are then classified on the basis of region localization through density-based spatial clustering of applications with noise (DBSCAN). However, these methods have limited detection accuracy when intensity values are uniform, and regions are smooth or flat. Keypoint-based methods generally exhibit high false-positive rates in images having natural similarity, and are not suitable for the detection of the duplication of smaller regions. These techniques perform well under postprocessing attacks such as rotation and scaling [23,26].

A hybrid technique combines block- and keypoint-based approaches. The approach presented in [27] is a hybrid of block- and keypoint-based feature extractions. Block-based extraction relies on Fourier–Mellin transform (FMT), whereas scale invariant feature transform (SIFT) is employed for keypoint feature extraction. SIFT features are extracted from textured regions, and FMT is applied on the smooth region. Matching ensuing features determines the duplication of blocks or regions.

### 2.3. Frequency-Domain Methods

We now discuss some frequency domain methods with special reference to discrete cosine transform (DCT) and DWT. The method in [28] divides the image into overlapping blocks and computes DCT coefficients. By using the signs of DCT coefficients, binary feature vectors are created and then matched using the coefficient of correlation. The method in [29] employs scale-invariant feature transform (SIFT) in combination to DCT. In [26], a cellular automaton was used to realise feature vectors based on DCT coefficients from the blocks into which the image is already divided. For duplication matching, the ensuing feature vectors are subjected to a K-dimensional tree (KD-tree)-based nearest-neighbor search. This method claims to be robust against common postprocessing attacks.

DWT is a popular transform for its localized nature and the ability to compress most image information into the lowest energy sub-band that is dyadically reduced in proportional size to the image. Hence, rather than the suspect image, its lowest energy sub-band can be subjected to forensic analysis to reduce complexity; a Level 2 sub-band would have had 16 times fewer coefficients to analyze. In addition, DWT may enable the extraction of very good and robust features for comparison. A DWT-based method [30], first exhaustively searches for the identification of matching blocks and then uses phase correlation for the detection of the copied region. However, the technique gives poor results if the copied region is slightly scaled or rotated. In [11], pixel matching and DWT techniques were utilized to reduce dimensions. Moreover, phase correlation was used for the detection steps in the copied and pasted regions. To improve forgery localization, mathematical morphology was employed for the connected regions. The above-mentioned technique has low complexity and exhibits robustness against the postprocessing of the copied regions. However, performance depends on the scene of the copy/move image.

Another copy–move forgery detection algorithm for color images is based on sensor pattern noise (SPN) [31]. Pattern noise is extracted by using the wavelet-based Wieners denoising filter. Features are selected on the basis of the signal-to-noise ratio, information entropy, variance in pattern noise, and average of energy gradient of the extracted image. This method is robust against geometric transformation (rotation and scaling), noise, and JPEG compression. The technique in [32] is based on DWT and DCT quantization coefficient decomposition (DCT-QCD). The method exhibits accuracy, but does not show robustness against rotation and scaling.

## 3. Proposed Method

**Figure 2:**
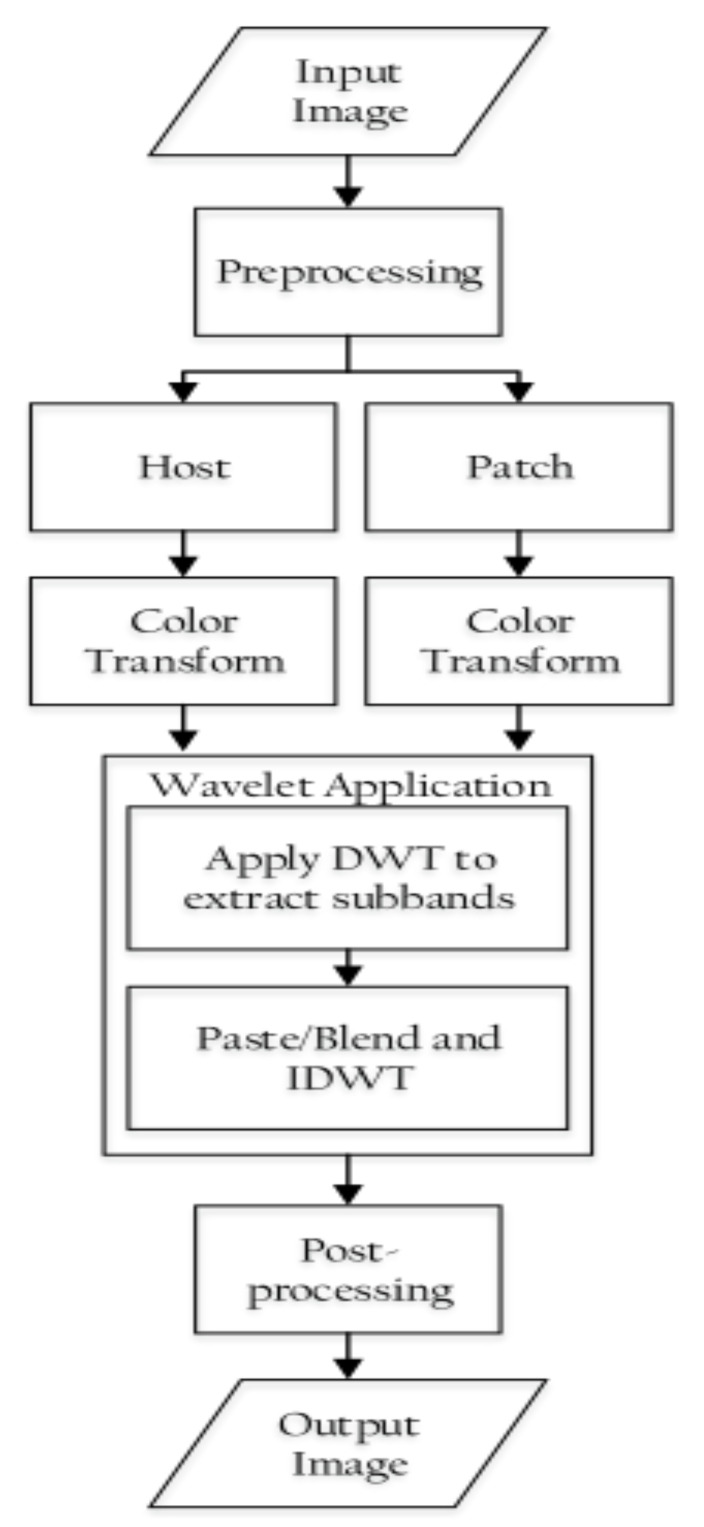
Block diagram representation of frequency-domain manipulation.

A block diagram outlining the proposed method is shown in Figure 2. The method involves the following steps:

### 3.1. Preprocessing

Before treating the image in the frequency domain, some preprocessing is inevitable. These include cropping the patch that is extracted from the original image and suppressing any noise in both host image (image A) and patch (image B) via smoothing.

### 3.2. Color Transformation

Before passing to the DWT domain, color transform (CT) is applied to both host and patch from RGB to the YCbCr domain to facilitate image manipulation in subsequent steps. The RGB to YCrCb conversion is also called irreversible color transform (ICT) because the process is not completely reversible and is suited to lossy compression schemes. It involves the following equation:

(1) $$\left[\begin{array}{c} Y\\ Cb\\ Cr \end{array}\right]=\left[\begin{array}{ccc} 0.299 & 0.587 & 0.114\\ -0.16875 & -0.33126 & 0.5\\ 0.5 & -0.41869 & -0.08131 \end{array}\right]\;\times \left[\begin{array}{c} R\\ G\\ B \end{array}\right]$$

For the lossless case, a very simplistic alternative, reversible color transform (RCT), is recommended, given by

(2) $$\left[\begin{array}{c} Y\\ Db\\ Dr \end{array}\right]=\left[\begin{array}{ccc} 1 & 2 & 1\\ 0 & 1 & -1\\ 1 & -1 & 0 \end{array}\right]\;\times \left[\begin{array}{c} R\\ G\\ B \end{array}\right].$$

### 3.3. Applying Wavelet Transformation

Level *l* DWT is applied to each of the YCbCr components of both A and B to obtain 3l+1 sub-bands for each of the YCbCr components. The size of a given sub-band is a dyadic fraction of the image size. For example, if l=1, then for a square image of dyadic size n×n, we obtain four sub-bands (LL, HL, LH, and HH), each of size n/2×n/2. The LL sub-band is the lowest-frequency sub-band containing most of the image’s energy. In other words, LL is the low-pass representation of the image zoomed out to n/2×n/2; the three other sub-bands, i.e., HL, LH, and HH, are the high-pass products in the horizontal, vertical, and diagonal directions, respectively. Wavelet transforms are usually characterized by symmetry, smoothness, and compactness in the form of filter length and orthogonality of the underlying wavelets. There are two practical ways to subject an input signal to DWT, namely, convolution and lifting. Due to its lesser computational complexity, lifting mode is usually preferred and separably employed, i.e., two 1D transforms one after the other rather than 1D at once. For simplicity, we chose Haar’s wavelet transform as our DWT. Let the 1D (pixel row or pixel column) input signal ($s_{0},s_{1},s_{2},\ldots ,s_{n-1}$), resulting in low-pass sub-band signal $L_{0},L_{1},L_{2},\ldots ,L_{n/2-1}$ and high-pass sub-band signal $H_{0},H_{1},H_{2},\ldots ,H_{n/2-1}$; after the DWT, a lifting-based Haar transform takes the following form:

(3) $$\left\{\begin{array}{ccc} L_{i} & = & \frac{s_{2i}+s_{2i+1}}{2},\\ H_{i} & = & s_{2i}-L_{i}\;\mathrm{or}\;H_{i}\;=\;L_{i}-s_{2i+1}. \end{array}\right.$$

### 3.4. Pasting or Blending and Applying Inverse DWT

While keeping into account the correspondence of both the components and their sub-bands, each sub-band of B is pasted to the identified place in the corresponding sub-band of A. This pasting may be carried out just by simple cut-out or alpha matting, or even using a gradient transfer such as Poisson image editing, as elaborated in Section 2. Level *l* inverse DWT is applied to the blended sub-bands from the last step to obtain the Y, Cb, and Cr components of the wavelet-transformed manipulated image.

### 3.5. Inverse Color Transformation

In the last step, all sub-bands of Y, Cb and Cr are combined to obtain the transformed YCbCr image. The resultant tampered image (A’) is obtained by passing from the YCbCr back to the RGB domain according to the following equation:

(4) $$\left[\begin{array}{c} R\\ G\\ B \end{array}\right]=\left[\begin{array}{ccc} 1 & 0 & 1.402\\ 1 & -0.34413 & -0.71414\\ 1 & -1.772 & 0 \end{array}\right]\;\times \left[\begin{array}{c} Y\\ Cb\\ Cr \end{array}\right].$$

The whole idea behind using the DWT domain pasting and subsequent inverse DWT is to dilute any artifacts that may especially result along the contours of the patch in its new environment in the host. Inverse DWT has the capacity to smooth such artifacts.

## 4. Experimental Results

We applied our method to a set of images from various sources from the Internet, and results were interesting when inspected and compared with two state-of-the-art methods from the literature.

### 4.1. Evaluation Metrics

The effectiveness of the forgery detection methods is usually gauged by two measures: detection accuracy (*r*) and false detection rate or FDR (*w*). These are computed by the following equations:

(5) $$r=\frac{|R\cap D|}{|R|}$$

(6) $$w=\frac{|F-D|}{|R|}$$

where *R* represents the actual tampered area, *D* is a detected area, and *F* is a falsely detected area.

### 4.2. Benchmark Methods

We chose two methods for the sake of comparison in order to judge the effectiveness of the proposed method. The first method is by Mahmood et al. [5] (Mahmood’s method). The second method is by Meena and Tyagi [6] (Meena’s method).

#### 4.2.1. Mahmood’s Method

This method detects image forgery by feature matching using Tchebichef moments [33], as the suspect image is first segmented into overlapping blocks, and Tchebichef moments are computed for every block. SVD is applied for dimensionality reduction, followed by lexicographic sorting that brings similar vectors the closest to each other. After morphological processing, classification is carried out on the basis of a preset threshold value. This technique is claimed to be capable of unveiling both single and multiple copy–move forgeries in the presence of postprocessing, such as brightness change, color reduction, contrast adjustment, compression, and blurring.

#### 4.2.2. Meena’s Method

In Meena’s method, the suspect image is divided into fixed-size overlapping blocks, and each block is subjected to Tetrolet transform [34] to extract low- and high-pass coefficients from each block. A lexicographic sort ensues on the basis of the four features of each block in order to check the similarity measure on the basis of a threshold value. The method is claimed to be robust against small and multiple forgeries, even in images that are scaled and those subjected to some postprocessing such as blurring, rotation, and brightness adjustments.

After dealing with preliminaries, we now present our results, with a simulation example first to elaborate the function of the method, and then the overall results.

### 4.3. Simulation Example

**Figure 3:**
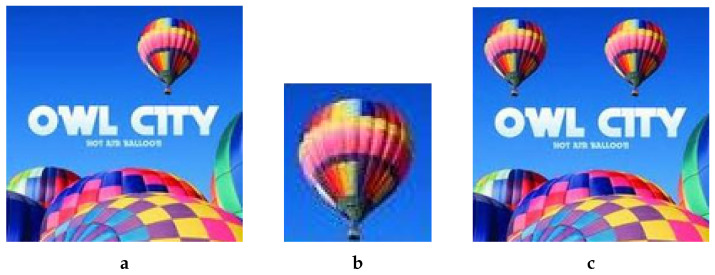
A simulation example of copy/move forgery. (**a**) Original/Host Image, (**b**) Patch Image, (**c**) Forged Image.

For demonstration, we take one example from our dataset. Figure 3 shows (A) example original image (http://www.famousfix.com/topic/owl-city-hot-air-balloon-album (accessed on 8 December 2021)) and (B) the corresponding patch image taken from the host image.

**Figure 4:**
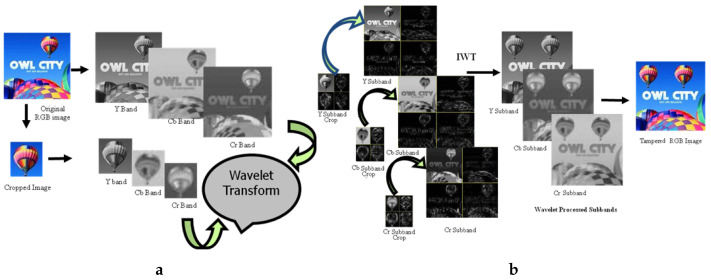
DWT domain tampering. (**a**) Transforming into DWT domain, (**b**) Copy/paste and subsequent IDWT.

The overall process for this specific manipulation is graphically illustrated in Figure 4. First, we cropped out a small region (hot air balloon) from the original image to serve as a patch to be pasted at a predetermined position in the host image. Since we were performing copy/move manipulation, the original image was also the host image. Figure 4a shows that both patch and host were converted into the YCbCr domain; subsequently, each resultant component was subjected to DWT. In this particular case, as Figure 4b shows, for illustration purposes, only a single-level DWT (l=1) was employed, which resulted in four sub-bands each for each of the three YCbCr components of both the host and the patch. However, on the basis of resolution, higher levels DWT are recommended for seamless results; with our experiments we mostly relied on level-2, which results in seven sub-bands. Each sub-band of the patch was pasted to its corresponding host sub-band of a given component to obtain the forged DWT domain *Y*, Cb and Cr components, as shown in Figure 4b. The application of inverse DWT resulted in the *Y*, Cb and Cr components of the forged image. These three components were combined to give the final forged RGB image shown in Figure 3c.

**Figure 5:**
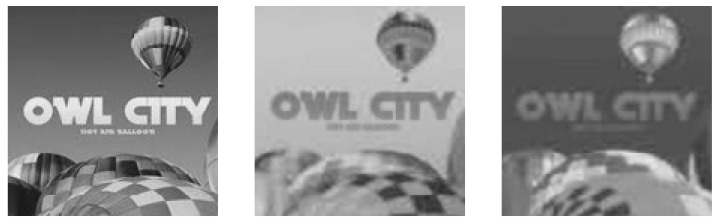
Luminance/chrominance components of original image.

**Figure 6:**
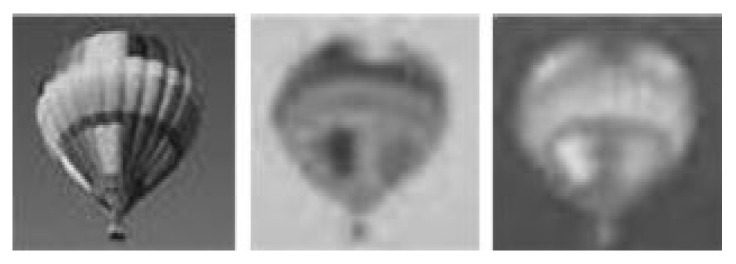
Luminance/chrominance (YcbCr) components of the patch.

For clarity, we presented results from each step one by one. The images were subjected to color transform in the shape of YCbCr to obtain the luminance component (Y) and the two chrominance components (Cb and Cr) for both host (Figure 5) and patch (Figure 6).

**Figure 7:**
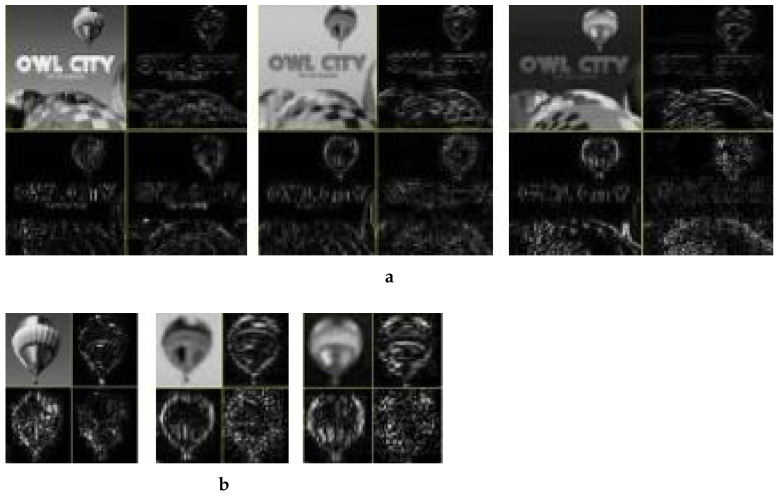
Level-1 wavelet-transformed YCbCr components. (**a**) Original Image, (**b**) Patch image.

Application of Level 1 Haar’s DWT resulted in four sub-bands (LL, HL, LH and HH) for both A and B, as shown in Figure 7. The LL sub-band (top left in each image) of each component was its low-pass version. The three other sub-bands were high-pass representations.

**Figure 8:**
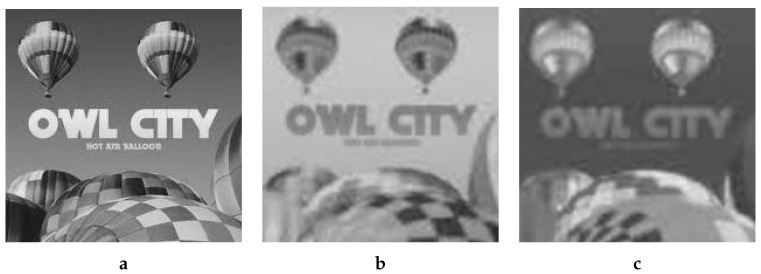
Luminance/Chrominance components of the forged image. (**a**) Y, (**b**) Cb, (**c**) Cr.

Keeping the correspondence in view, the LL sub-band from the DWT-ed luminance component of B was pasted in the LL sub-band from the DWTed luminance component of A. In a similar fashion, the HL, LH and HH sub-bands from the DWTed luminance component of B are pasted in the HL, LH and HH sub-bands from the DWTed luminance component of A, respectively. The same procedure was adopted for the DWT domain sub-bands of the two chrominance components. After pasting the patch sub-bands to the corresponding sub-bands of host, we obtained the forged image in DWT domain, which was subjected to inverse DWT to obtain the YCbCr components shown in Figure 8.

Applying inverse color transform to YCbCr resulted in RGB image (A’) in Figure 3c.

### 4.4. Overall Results

**Figure 9:**
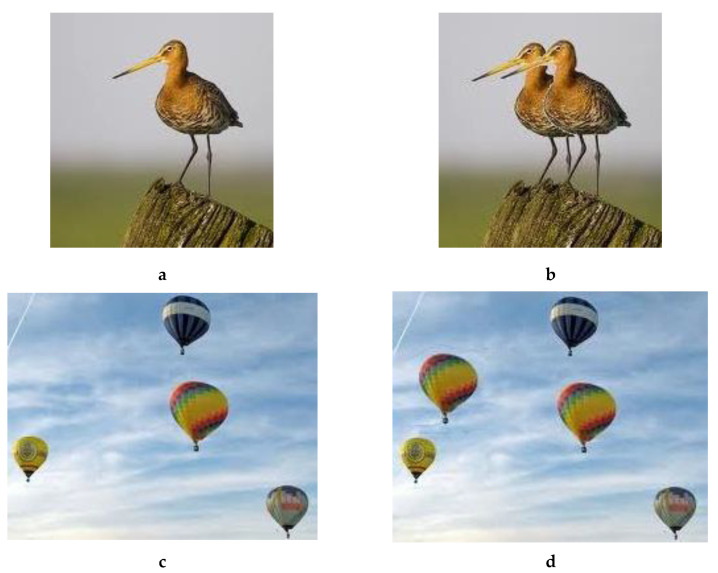
Example Results. (**a**) Original, (**b**) Forged, (**c**) Original (https://upload.wikimedia.org/wikipedia/commons/e/e9/WIM_2004_balloons.jpg (accessed on 8 December 2021)), (**d**) Forged.

The simulation result above shows that it is hard to discern tampering with a naked eye, especially in the absence of the original. For illustration purposes, we show results of two more examples in Figure 9. However, one cannot solely rely on subjective results; therefore, it is t imperative to test the effectiveness of the proposed method against effective methods from the literature. We thus relied on two methods, i.e., the Mahmood and Meena methods. The average results over the set of all test images manipulated by the proposed method after subjecting to the two reference detection methods are shown in Table 1. With Mahmood’s method, average detection accuracy was as low as 14.72%, and with Meena’s method, it was even worse, i.e., 17.27%. The FDR was also considerably high for both the methods and could not be rejected as insignificant. Thus, Mahmood’s method failed to detect the forged regions and only detected naturally similar blocks in the tampered image. Similarly, the detection results of the Tetrolet transform (Meena method) were also not enviable.

We created a large set of images with our method, but for illustration purposes, we relied on two representative examples here: (1) forged balloon image with non-overlapped copy/move operation (Figure 3c), and (2) forged bird image where the copied area partially hides or overlaps with its origin (Figure 9b). This type of forgery is normally considered to be harder to detect.

**Figure 10:**
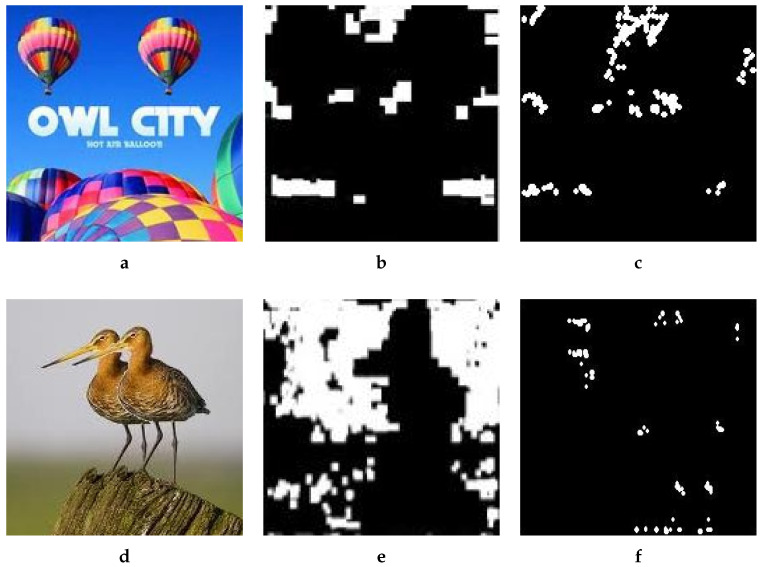
Forgery detection results by two benchmark methods. (**a**) Forged, (**b**) Mahmood, (**c**) Meena, (**d**) Forged, (**e**) Mahmood, (**f**) Meena.

Figure 10 demonstrates the low detection accuracy of the two benchmark methods with two representative examples. For comparison, we also included the forged images in Figure 10a,d. We performed a simple copy–move operation without any postprocessing such as rotation, scaling, noise addition, and JPEG compression, yet not a single suspect block was detected in the forged regions, and the false detection rate was almost high in both images. Experimental results in Figure 10b,e reveal that Mahmood’s method only detected naturally similar blocks from the forged image and gave no clues about the forged regions. Results with Mahmood’s method are given in the form of binary images in Figure 10b,e wherein black regions show the unsuspected parts, whereas white regions indicate suspected ones. Suspected parts may pertain to an actually forged or falsely detected region, or the natural similarity of the pixels. In the two images, the fraction of white pixels was too low and within the range of natural similarity.

Meena’s method also showed similarly lower detection accuracy, shown in Figure 10c,f. There were not enough clues to detect the forged area. The method relies on plain copy–move forgery, i.e., part of the image is copied or replicated in a same image without applying any type of postprocessing. As the image shows in Figure 10c,f, the detection accuracy stood at almost zero and gave more potency to forgery detection algorithms. Therefore, the results of Meena’s method reveal that the accuracy rate of the forged regions was almost untraceable, and only natural similarity is detected.

**Figure 11:**
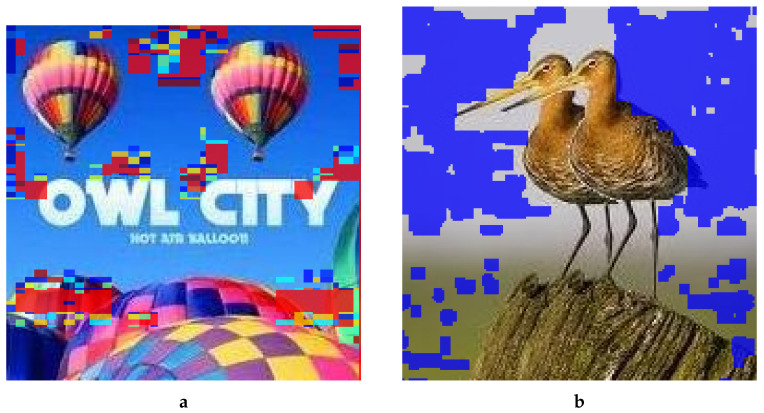
Forgery detection results provided by the authors of Mehmood’s method. (**a**) Forged balloon image, (**b**) Forged birds image.

**Figure 12:**
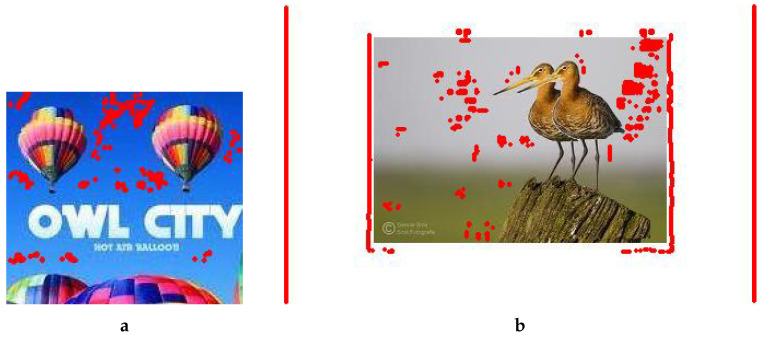
Forgery detection results provided by the authors of Meena’s method. (**a**) Forged balloon image, (**b**) Forged birds image (the copyright logo is visible on the tested forged version; for better viewing, a cropped version of the image is used in Figure 5a).

We are thankful to the authors of both benchmarking methods [5,6] for accepting our request to employ their method to test some of our manipulated images. They provided us with the output of their method in the form of the images in Figure 11 and Figure 12. The first author of Meena’s method commented, “As you can see our method could not detect the forgery properly in either of the images that you have provided. This may be due to high JPEG compression. As we have tested this method on JPEG compression up to quality factor 80, beyond that our Tetrolet-based CMFD may fail”. Let us emphasize that we had not applied any compression to the images in any form, and this reveals the success of our method.

**Figure 13:**
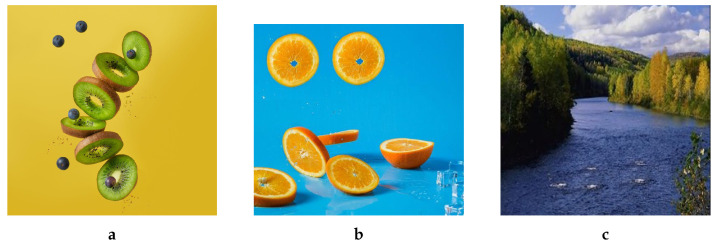
Examples of additional forged images along with the sizes (The first two images derived from images found on https://unsplash.com/ (accessed on 9 March 2022) while the third one derived from an image taken from CASIA dataset [35]). (**a**) 1024 × 1024, (**b**) 502 × 457, (**c**) 256 × 256.

Three additional images that were forged by the proposed method are presented in Figure 13 along with their sizes. For the sake of visual demonstration, the corresponding original host images are withheld.

## 5. Conclusions and Future Work

By tampering with images in the DWT domain and subsequently applying the inverse DWT, we were able to obtain robust results. The presumption that the inverse DWT has enough potential to do away with artifacts or side effects resulting from any manipulation proved to be valid, at least with copy/move forgeries. As the experiments suggest, the two detection methods were not that successful in zeroing in over tampered areas, and for that reason, there is a need to improve and refine the forgery detection methods.

As a future perspective, investigations can be carried out to combine the proposed method with state-of-the-art image compositing techniques, especially gradient-based methods such as Poisson image editing. In addition, the success of the method necessitates to go beyond the domain of copy/move manipulation and explore the scenario in which the patch is not taken from the host image. 

## Figures and Tables

**Table 1:** Result Metrics.

Method	Detection Accuracy	FDR
Mahmood’s method [5]	14.72%	29.14%
Meena’s method [27]	17.27%	22.13%

## Data Availability

Please contact the first author by email; The data will be made formally available on GitHub in due course.
